# Chronic Subdural Hematoma in Patients Over 90 Years Old in a Super-Aged Society

**DOI:** 10.14740/jocmr1907w

**Published:** 2014-07-28

**Authors:** Sadaharu Tabuchi, Mitsutoshi Kadowaki

**Affiliations:** a Department of Neurosurgery, Tottori Prefectural Central Hospital, Tottori, Japan

**Keywords:** Chronic subdural hematoma, Surgical treatment, 90 years old, Aged, Elderly

## Abstract

**Background:**

Chronic subdural hematoma (CSDH) is one of the most common diseases in neurosurgical practice, particularly among aged patients. With the continuing increase in the aged population, further increases in incidence are expected. However, few studies have focused on CSDH in super-aged patients over 90 years old.

**Methods:**

We retrospectively reviewed medical records for 20 consecutive patients over 90 years old with CSDH treated in our department between 2007 and 2013. The diagnosis of CSDH was confirmed by computed tomography (CT). Patients were divided into a surgery group and a conservative group. Surgical procedures included burr-hole surgery followed by insertion of a subdural drain under local anesthesia. Clinical data were compared and analyzed. Neurological status was evaluated according to the modified Rankin Scale at three time points: before suffering from CSDH; at the time of referral or admission to our department; and at discharge or 1 month after the first referral. Statistical tests were used to analyze data and values of P < 0.05 were considered significant.

**Results:**

Mean age for the 20 cases was 92.6 years (range, 90 - 96 years). The leading symptoms in this population were hemiparesis and gait disturbance, followed by disturbance of consciousness and speech disturbance. Twelve patients underwent burr-hole surgery. Mean maximum thickness of subdural hematoma as measured on CT was significantly higher in the surgery group (28.2 ± 5.4 mm) than in the conservative group (17.0 ± 3.8 mm; P < 0.01). Postoperatively, mean neurological status was significantly improved in the surgery group (P < 0.01). After surgery, 66.7% of patients could return home directly from hospital. No significant perioperative complications directly related to surgery were encountered in the surgery group, except for transient postoperative restlessness and bruising of extremities due to falls.

**Conclusions:**

Surgery for CSDH is safe and positively recommended even in super-aged patients over 90 years old if the patient’s physical status is fair. Pre-illness status is the most important factor for considering operative indications and represents a limiting factor for postoperative outcomes in this age population.

## Introduction

Chronic subdural hematoma (CSDH) is one of the most common diseases encountered in neurosurgical practice, particularly among aged patients. As few studies have reported on the treatment of CSDH in super-aged patients over 90 years old, we analyzed the data available from patients we have treated and discussed the problems characteristic to this population.

## Materials and Methods

We retrospectively reviewed 20 consecutive patients with CSDH aged over 90 years old treated in our department between 2007 and 2013. The diagnosis of CSDH was confirmed by computed tomography (CT). Fifteen patients suffered from unilateral CSDH, and five patients showed bilateral CSDH. Patients were classified according to treatment into a surgery group and a conservative group. Clinical data concerning hematoma location, maximum thickness of hematoma, past history, usage of antiplatelet and/or anticoagulant drugs, and outcomes were analyzed. Treatment was selected based on several factors, including physical status of the patient, severity of symptoms, mass effect on CT, and the wishes of the patient and/or their family. Neurosurgical treatment was performed under local anesthesia. The procedure involves a single burr-hole craniostomy and irrigation. Subdural hematoma was evacuated by irrigation with physiological saline solution followed by placement of a closed-system subdural drain for 1 - 2 days. The duration of drainage depended on the amount of subdural hematoma remaining as verified by CT. Since 2013, commercially available artificial cerebrospinal fluid (Artcereb; Otsuka Pharmaceutical Factory, Tokushima, Japan) [1] has been used instead of physiological saline for reasons of safety and improved prevention of recurrence.

Neurological status was evaluated according to the modified Rankin Scale (mRS) at three time points: before suffering from CSDH (based on interview with the patient’s family or acquaintance); on referral/admission to our department; and at the time of discharge or 1 month after first referral in cases of conservative treatment.

Statistical analysis was performed using Student’s non-paired *t*-test (*t*-test), Pearson’s Chi-square test and the Wilcoxon/Kruskal-Wallis test. Differences were considered significant for probability values of P < 0.05.

## Results

Mean age for all 20 patients was 92.6 ± 1.9 years (range, 90 - 96 years), including 11 men (55%) and nine women (45%). Patient characteristics are described in Table 1. Twelve patients underwent surgery for CSDH. Mean maximum thickness of subdural hematoma as measured on CT was significantly higher in the surgery group (28.2 ± 5.4 mm) than in the conservative group (17.0 ± 3.8 mm; P < 0.01). Other factors showed no significant difference between groups (Table 1). At the time of diagnosis, antiplatelet and/or anticoagulant medicines were being used by three patients (25%) in the surgical group and four patients (50%) in the conservative group. The most common concomitant disease was hypertension (45%), followed by dementia (35%) and cerebral infarction (30%). Notably, dementia was a significant factor in this aged population and limited the outcomes.

**Table 1:** Clinical Characteristics of 20 Patients With CSDH

	Surgery	Conservative	Significance	Statistics
Gender			NS	Chi-square test
Male n (%)	6 (50.0)	5 (62.5)		
Female n (%)	6 (50.0)	3 (37.5)		
Mean age years ± SD	92.2 ± 1.8	93.1 ± 2	NS	*t*-test
Hematoma location			NS	Chi-square test
Left n (%)	5 (41.7)	3 (37.5)		
Right n (%)	5 (41.7)	2 (25.0)		
Bilateral n (%)	2 (16.6)	3 (37.5)		
Maximum thickness (mm) ± SD	28.2 ± 5.4	17.0 ± 3.8	*P < 0.01	*t*-test
History of head injury n (%)	5 (41.7)	5 (62.5)	NS	Chi-square test
Past history of dementia n (%)	5 (41.7)	2 (25.0)	NS	Chi-square test
Antiplatelet/coagulation therapy n (%)	3 (25.0)	4 (50.0)	NS	Chi-square test

n: number of cases; SD: standard deviation; NS: not significant.

The present clinical symptoms are described in Table 2. The leading symptoms in this population were hemiparesis and gait disturbance, followed by disturbance of consciousness and speech disturbance. Unlike populations of other ages, no patients complained of headache.

**Table 2:** Presented Clinical Symptoms

Symptoms n (%)	Surgery	Conservative
Hemiparesis/gait disturbance	9 (75.0)^$^	3 (37.5)
Consciousness disturbance	3 (25.0)^$^	4 (50.0)
Speech disturbance	3 (25.0)^$^	0 (0.0)
Urinary incontinence	1 (8.3)	0 (0.0)
Absence of symptoms	0 (0.0)	1 (12.5)

^$^: duplication of symptoms.

**Figure 1:**
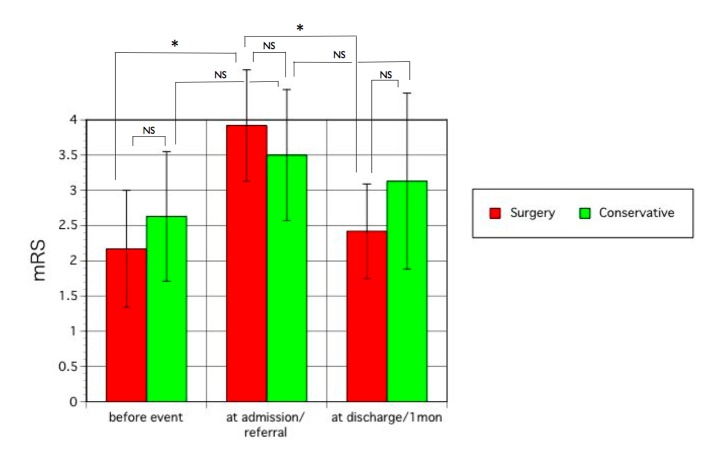
Neurological status as evaluated by mRS before the event (CSDH), on admission or referral to hospital, and at discharge after surgery or 1 month after treatment in the surgery group and conservative group, respectively. Statistical analysis was performed using the Wilcoxon/Kruskal-Wallis test and *t*-test. Error bars show standard deviation. NS, not significant. *P < 0.01.

Mean mRS before the event, on admission/referral, and at discharge/1 month later are shown in Figure 1. After surgery, mean neurological status was significantly improved in the surgery group (P < 0.01), but was unchanged in the conservative group after treatment. Neurological status at discharge did not differ from status before onset of CSDH in the surgery group (Fig. 1).

The mortality rate of acute hospitalization was 0% in the surgery group and 12.5% in the conservative group due to general complications resulting from poor physical status. Significant perioperative complications directly related to surgery were fortunately not encountered in the surgery group, with the exception of transient postoperative restlessness and bruising of extremities due to falls. After surgery, all patients showed clinical improvement at discharge and 66.7% of patients were able to return home directly from hospital. Recurrence of subdural hematoma requiring surgery was not observed in the surgery group during the follow-up period, but we cannot rule out the possibility that some patients may have been lost to follow-up, meaning that they were not seen in the outpatient clinic after the several hospital visits, and may have experienced recurrence and been referred to another hospital or died. The real incidence of recurrence might thus be difficult to evaluate in this elderly population.

## Discussion

In a super-aged society, CSDH is one of the most common diseases encountered in neurosurgical practice, and is easily treated with burr-hole surgery and subdural drainage. The incidence of CSDH increases with age, with a recent estimated incidence of 20.6/100,000/year for patients between 70 and 79 years old and 127.1/100,000/year in those over 80 years old [2]. With continuing increases in the aged population, further increases in incidence are expected and CSDH is becoming a typical disease of aged patients in most neurosurgical hospitals.

In Japan, the population over 90 years old comprises 1.13% according to Japanese national census data published in 2011 by the Ministry of Health and Welfare of Japan. We calculated the proportion of the population over 90 years old in Tottori Prefecture, as a representative advanced aging society with fewer children in Japan, from the estimated age-specific population data for Tottori Prefecture reported on the official website of the Tottori Prefectural Government in 2013, and found that the rate was 2.02%.

Few studies have investigated the issues associated with CSDH in super-aged patients over 90 years old [3]. The overall outcome in our study was relatively favorable, contrary to expectations.

The baseline activities of daily living (ADL) significantly limit the outcomes, particularly in patients with dementia. After surgery, the neurological status of all patients was significantly improved. Patients with dementia were able to walk after surgery, but ADL will still be limited in these patients, who will remain dependent. After surgery, 66.7% of patients in this study could return home, unlike previous reports [3]. In another study, Fukui et al reported that 89% of patients with CSDH showed improved status at the time of discharge, suggesting that surgical procedures represent the best choice for treating CSDH, even in extremely aged patients [4], consistent with our results.

The consensus opinion is that head trauma is the most common cause of CSDH. The incidence of falls peaks in both males and females between 75 and 79 years old [5]. In the present study, the frequency of a history of head trauma was relatively low, although this might have been influenced by patients with dementia and their families not remembering or noticing minor traumas, or in cases of patients living alone, episodes may not have been observed by others.

The leading symptom was hemiparesis associated with/without gait disturbance, which significantly worsened the ADL. Consciousness disturbance and speech disturbance such as aphasia were the next most common symptoms. All patients with consciousness disturbance in the surgery group were living alone, resulting in delayed recognition of symptoms and referral to hospital. Disturbance of consciousness in the conservative group was a transient symptom induced by other causes unrelated to the CSDH. In such patients, the CSDH was found incidentally on CT, and diagnosed as asymptomatic CSDH. Syncope or transient loss of consciousness is a common symptom in super-aged patients. This tendency might represent a unique finding in this elderly population.

The rate of perioperative complications was low. This may be related to appropriate preoperative evaluation and screening of patients’ general condition, meaning that patients with poor physical status were not scheduled to undergo surgery. Mortality rates associated with burr-hole surgery for CSDH reported in the literature range from 0% to 32% [6]. Stippler et al reported that nearly 31% in the surgical group aged 90 years or older were sent to a hospice or died, compared to none of the patients in the conservative group [3], diverging markedly from our results. However, Miranda et al mentioned that survival was unrelated to the type of surgical treatment or whether surgery was performed [7]. Borger et al stated that older age was unrelated to increased mortality in their series [8]. These findings are consistent with our results.

Considering the results of this study, super-aged patients may experience good functional recovery after treatment with burr-hole surgery. Interestingly, paradoxical results after surgery for CSDH have been reported in which patients over 80 years old experienced greater overall improvement of neurological function than those aged 70 - 79 years old [9]. Another study about the surgical treatment of CSDH reported that age had no influence on outcomes for patients 80 years old or more compared to patients less than 80 years old [10]. Recently, a substantial proportion of patients over 80 years old have been living alone or as an elderly couple in local districts in Japan, with ADL at a level allowing independent living. Paradoxically, the population over 90 years old may generally have less severe concomitant disease, although the rate of dementia is high. Patients with severe general concomitant diseases may tend to die before reaching 90 years old. That is, the population over 90 years old may have passed through a process of natural selection of weak individuals in terms of survivability. This tendency may strengthen the possibility that super-aged patients with CSDH may possess substantial recovery potential and could receive benefits from burr-hole surgery.

Careful decision-making is necessary in patients suffering from CSDH using antiplatelet and/or anticoagulant therapy, because antiplatelet and anticoagulant therapies are more prevalent among non-traumatized CSDH patients with cerebrovascular/cardiovascular co-morbidities [11] and are significantly associated with increased risk of CSDH [12].

A key limitation of this study was the small overall number of patients over 90 years old and little information is available about patients in their late 90s and beyond. Patients were not randomly assigned to receive surgical procedures or conservative treatment due to the retrospective nature of the study. The beneficial effects of surgery would presumably have been affected by selection bias, with healthier patients being offered surgical treatment. In addition, long-term outcomes were not assessed in this study, and should be examined in the future.

### Conclusion

Surgery for CSDH is safe and can be positively recommended even for super-aged patients over 90 years old if the physical condition of the patient is fair and operative indications are appropriate. Pre-illness status represents the most important factor when considering operative indications and represents a limiting factor for postoperative outcomes in this elderly population.
